# Impact of Thyroid Autoimmunity in euthyroid women on the outcomes of In Vitro Fertilization

**DOI:** 10.1016/j.amsu.2021.102473

**Published:** 2021-06-08

**Authors:** Ahed Hamad, Nawras Alhalabi, Nazht Nmr, Fatima Abbas, Hisham Al-Hammami, Nazir Ibrahim, Marwan Alhalabi

**Affiliations:** a Faculty of Medicine, Damascus University, Damascus, Syria; b Al-Zahrawi Maternity Hospital, Damascus, Syria; c Faculty of Medicine, Syrian Private University, Damascus, Syria; d Department of Obstetrics and Gynecology, Faculty of Medicine, Syrian Private University, Damascus, Syria; e Department of Internal Medicine, Faculty of Medicine, Syrian Private University, Damascus, Syria; f Division of Reproductive Medicine, Embryology and Genetics, Faculty of Medicine, Damascus University, Damascus, Syria; g Assisted Reproduction Unit, Orient Hospital, Damascus, Syria

**Keywords:** In vitro fertilization (IVF), Intra cytoplasmic sperm injection (ICSI), Thyroid autoimmunity (TAI), Clinical pregnancy rate (CPR), Syria

## Abstract

**Objective:**

Infertility is inadequately acknowledged as a problem in developing countries. Thyroid Autoimmunity (TAI) has been neatly investigated for its association with unfavorable pregnancy and reproductive consequences. We aim to evaluate Clinical Pregnancy Rate (CPR) as a primary outcome following In Vitro Fertilization/Intra Cytoplasmic Sperm Injection (IVF/ICSI) in women with Thyroid Autoimmunity (TAI).

**Methods:**

A Retrospective cohort study included 584 women who underwent IVF/ICSI treatment between November 2012 and April 2017 in Orient Hospital, Damascus, Syria. Patients were tested for TAI before IVF/ICSI procedure.

**Results:**

CPR did not significantly differ between TAI positive and TAI negative groups (p > 0.05). Subgroup analysis for only primary infertility patients showed a statistically significant difference in CPR between TAI positive and TAI negative groups.

**Conclusion:**

Although several arguments were in favor of the relation between IVF/ICSI outcomes and Thyroid autoimmune disease, the presence of TAI positivity did not adversely affect the clinical pregnancy rate.

## Highlights

• Thyroid Autoimmunity has been investigated for its association with unfavorable pregnancy and reproductive consequences.
• The prevalence of TAI in Syrian women having IVF/ICSI is 25.3%.
• The positivity of Thyroid Autoimmunity did not adversely affect the clinical pregnancy rate for women underwent IVF/ICSI.
• Screening of Thyroid Autoimmunity constitutes a financial burden on patients more than its benefit.
• The findings highlight the infertility aspects of the Syrian population.

## 1 Background

Infertility is inadequately acknowledged as a problem in developing countries [1]. Since it was first introduced in 1978, in vitro fertilization-embryo transfer (IVF-ET) still is the most commonly used technique of assisted reproduction with increasing success rates [2]. Thyroid Autoimmunity (TAI) has been neatly investigated for its association with unfavorable consequences for pregnancy in all trimesters such as implantation failure, miscarriage, placental abruption, preterm birth, and increased risk of perinatal mortality [3,4].

Previous studies showed that 5–20% of women of childbearing age are affected by Thyroid Autoimmunity (TAI) which is characterized by the presence of anti-thyroid peroxidase (*anti*-TPO) and/or anti-thyroglobulin (*anti*-TG) antibodies [5]. Thyroglobulin (TG) is a molecule synthesized by thyroid cells to produce and store thyroid hormones. Thyroid peroxidase (TPO) is an enzyme involved in the production of thyroid hormones by iodination of surfactant protein A tyrosine residues [6].

TAI diseases are often underdiagnosed because they may be present without overt thyroid dysfunction for several years [7,8]. Accordingly, TAI is more prevalent in women attending fertility clinics compared to the general population [9,10]. However, the relationship between the presence of TAI and infertility continues to be broadly speculative [11]. A previous study investigated the impact of *anti*-TPO on reproductive biology and concluded that positive *anti*-TPO adversely affects folliculogenesis, spermatogenesis, fertilization rates (FRs), embryo quality, and pregnancy rates [12]. Moreover, when compared to controls, pregnancy outcomes in women with TAI subjected to In Vitro Fertilization (IVF) were lower [13].

In this study, we aim to evaluate the impact of TAI on IVF/ICSI outcomes such as Clinical Pregnancy Rate (CPR) as a primary outcome. To the best of our knowledge, this is the first study in Syria to investigate TAI in women undergoing IVF/ICSI.

## 2 Methods

This was a retrospective cohort study carried out using the computerized database at Assisted Reproduction Unit, Orient Hospital, Damascus, Syria. Data were manually checked for any mistakes in thoroughness and accuracy before initiating the study. Orient Hospital is a professional unit affiliated to the Faculty of Medicine, Damascus University, and an approved training facility for Assisted Reproduction Technology (ART) by the Ministry of Health in Syria. Ethical approval was obtained from the ethical and research committees in both Faculty of Medicine, Damascus University and Syrian Private University with the approval of Orient Hospital Board of Directors. The study was conducted in compliance with the STROCSS criteria [14]. Our work was submitted with a research Registry UIN: “researchregistry6786”.

### 2.1 Patients

All women undergoing IVF/ICSI cycles between November 2012 and April 2017 were tested for thyroid function, including Anti Thyroid Peroxidase Antibodies (*anti*-TPO), Anti Thyroglobulin Antibodies (*anti*-TG), Thyroid Stimulating Hormone (TSH), all of which were requested by the referring physician before ovarian stimulation.

### 2.2 IVF procedure

Women were treated either with the long Gonadotropin-releasing hormone (GnRH) agonist protocol or GnRH antagonist protocol for pituitary downregulation. The details for both protocols were previously described [15,16]. They received either (human menopausal gonadotropin) HMG or recombinant follicular stimulating hormone (rFSH) for ovarian stimulation following the menstrual period with dosage adjustment according to the response.

Transvaginal ultrasonography along with measurement of blood estradiol was used to assess the ovarian response. When 3 leading follicles reached 17–18 mm, we administered 10,000 IU of Human chorionic gonadotropin (HCG), and oocytes retrieval was done 35–36 h later. Fertilization was carried out in vitro by ICSI. In the cleavage stage (Day 3), at most three embryos were transferred with ultrasound guidance. Luteal phase support was achieved with vaginal micronized progesterone. Correspondingly, clinical pregnancies were confirmed by positive urine HCG tests and transvaginal ultrasonographic evidence of a gestational sac.

### 2.3 Collection of clinical information

Clinical information that was collected included age, place of residence, assessment for infertility type (primary or secondary), duration of infertility [17], history of undergoing assisted reproduction, and IVF indications. In addition, the data included tests recorded before ovarian stimulation in IVF/ICSI cycle such as Basal Follicular Stimulating Hormone (Basal FSH), Day 2 Estradiol (E2) and Anti Müllerian Hormone (AMH).

During the IVF treatment, data recorded included stimulation protocol, stimulation drug, sperm collection procedure, endometrium thickness, number of oocytes retrieved, M − II oocytes, fertilization rate, number of transferred embryos, and pregnancy rate.

### 2.4 Determination of antibodies and hormone levels

All tests were done in the same hospital lab. Lab Tests were primarily measured on the instrument Cobas 6000 (Roche Diagnostics). The instrument is a closed system, random access auto-analyzer. Performance characteristics of measurement are previously explained [18]. Particularly, two lots of calibrators were used in each instrument, four lots of reagents for Cobas were used during the procedures. Method calibrations were done on the instrument roughly once every month for the change of lots. Two levels of 3rd party commercial control materials were run for every working day.

Lab tests cutoff values were as follows: TPO antibodies ≥35 IU/ml, TG antibodies ≥40 IU/ml, TSH (0.45–4.5) uIU/ml, Basal FSH ≥ 12.5 mIU/ml, Day 2 Estradiol (E2) ≥ 50 pg/ml and AMH ≥ 1.5 ng/ml.

### 2.5 Data analysis

Only women with normal TSH levels were included in the study (0.45–4.5 μIU/ml). The primary outcome measure was defined as the Clinical Pregnancy Rate. Thyroid autoimmunity status was considered positive in the presence of *anti*-TPO and/or *anti*-TG higher than the upper limit of the reference range.

Subgroup analysis was done for a stricter TSH threshold of <2.5 [19,20], for prognosis related to age (Blue for less than 40 years and Red for 40 years and higher), low and normal AMH levels as an indicator for ovarian reserve, in addition to normal and poor ovarian reserve according to FSH (considering Day 2 Estradiol levels). The pregnancy rate was calculated as per completed embryo transfer.

Statistical analysis was done using the Statistical Program for Social Sciences (Version 25; SPSS Inc., Chicago, IL, USA). Mann–Whitney *U* test, Kruskal–Wallis H test, chi-squared test, and Fisher's exact test were used as appropriate. The cutoff value of p < 0.05 was considered statistically significant.

## 3 Results

A total of 584 patients who underwent 954 IVF/ICSI cycles were included in the study.

The prevalence of positive TAI was 148 patients (25.3%) on their first visit to our center. The mean age was 34.25 ± 5.601 years (mean ± SD) for TAI positive patients compared with 33.8 ± 6.143 years for the TAI Negative. Sixty four percent (n = 374) of patients were residents of Damascus Metropolitan area or Rural Damascus governorate, 25.0% (n = 146) lived in all other governorates of Syria and 11.0% (n = 64) lived abroad (mostly in Iraq). There was no significant difference in TAI positivity between places of residence. 32.9% of our patients were older than 40 years of age at the start of the cycle. No statistical difference was observed in TAI positivity between both groups of age. Demographic data are summarized in Table 1.

**Table 1:** TAI prevalence according to patients' demographics and cycles characteristics.

	TAI Positive		Total (% of cycles)		p Value
Patients' No.*	148	25.3%	584		
Age*	34.25	±5.601	34.19	±6.007	NS
Place of residence*, n (%)					
Damascus and Rif Dimashq	91	24.3%	374	64.0%	NS
All other Syrian governorates	40	27.4%	146	25.0%	
Outside Syria	17	26.6%	64	11.0%	

IVF Cycles No.	250	26.2%	954		
Age group					NS
Blue < 40	156	26.2%	595	62.4%	
Red > 40	94	26.2%	359	37.6%	
Infertility Type					NS
Primary	218	25.9%	841	88.2%	
Secondary	32	28.3%	113	11.8%	
Infertility Duration (years)					NS
0 - 4	78	27.9%	280	29.4%	
5 - 9	94	25.7%	366	38.4%	
10 - 14	49	28.8%	170	17.8%	
> 15	29	21.0%	138	14.5%	
IVF Indication					*p* < 0.05
Male Factor	90	23.2%	388	40.7%	
Female Factor	107	26.4%	405	42.5%	
Combined	31	40.3%	77	8.1%	
Unexplained	4	18.2%	22	2.3%	
PGD	18	29.0%	62	6.5%	
IVF History					NS
< 2	157	30.3%	518	54.3%	
2 - 3	65	21.0%	309	32.4%	
4 - 5	22	24.4%	90	9.4%	
≥ 6	6	16.2%	37	3.9%	

NS: non-significant.

Regarding IVF/ICSI procedures, 250 patients (26.2%) tested positive (per cycle) for TAI prior to IVF/ICSI cycle initiation. The sample was divided according to IVF/ICSI indication into 405 cycles (42.5%) for female factor infertility, 40.7% (n = 388) for male factors, 8.1% (n = 77) for combined male and female infertility causes, and 2.3% (n = 22) referred for unexplained infertility (Table 1).

Patients’ infertility duration at cycle initiation was between 0 and 4 years for 290 cycles (29.4%), 5–9 years for 366 (38.4%), 10–14 years for 170 (17.8%) and >15 years for 138 cycles (14.5%). In addition, 54.3% (n = 518) had a history of having only one or no prior IVF/ICSI cycles, 32.4% (n = 309) underwent 2 to 3 cycles, and 13.3% (n = 127) underwent 4 or more cycles (Table 1).

TAI positivity was significantly more prevalent in women undergoing IVF/ICSI for combined factors compared with other indications (40.3%, p < 0.05), no statistical difference in TAI status was found when comparing cycles concerning age group prognosis, infertility type, infertility duration, and history of IVF/ICSI procedures (Table 1).

Most of the cycles were carried out using long protocol IVF/ICSI method 84.5% (n = 799). There was no significant difference in TAI status regarding stimulation protocol or stimulation drug.

The response to ovarian stimulation in terms of endometrium thickness at the day of oocyte trigger, the numbers of oocytes retrieved, M − II oocytes, fertilization rate, and the number of transferred embryos were not statistically different according to TAI status (Table 2). In addition, similar results were found when subgrouping the data to TSH <2.5 only (Table 2).

**Table 2:** Initial IVF outcomes according to TAI positivity in Low and High Normal TSH subgroups.

	TSH <2.5					TSH <4.5				
	TAI Positive		Total (%)		*p* value	TAI Positive		Total (% of cycles)		p value
Endometrium (n = 909)	9.77	±5.60	9.67	±5.57	NS	9.47	±4.68	9.70	±6.05	NS
Oocytes Retrieved (n = 930)					NS					NS
0 - 4	42	24.3%	173	26.9%		60	26.4%	227	24.4%	
5 - 9	56	27.1%	207	32.2%		79	27.8%	284	30.5%	
≥ 10	64	24.3%	263	40.9%		107	25.5%	419	45.1%	
MII (n = 907)					NS					NS
0–25%	6	37.5%	16	2.5%		12	41.4%	29	3.2%	
25%–50%	15	22.1%	68	10.8%		23	22.1%	104	11.5%	
50%–75%	88	28.8%	306	48.7%		133	28.5%	466	51.4%	
75%–100%	47	20.0%	235	37.4%		70	22.7%	308	34.0%	
Fertilization Rate (n = 865)					NS					NS
0–25%	30	23.6%	127	21.2%		51	27.6%	185	21.4%	
25%–50%	57	25.3%	225	37.6%		88	25.0%	352	40.7%	
50%–75%	46	27.5%	167	27.9%		69	29.5%	234	27.1%	
75%–100%	15	18.8%	80	13.4%		17	18.1%	94	10.9%	
Transferred embryos (n = 866)					NS					NS
0	9	32.1%	28	4.7%		12	34.3%	35	4.0%	
1	22	27.5%	80	13.3%		37	32.7%	113	13.0%	
2	20	17.7%	113	18.8%		29	19.0%	153	17.7%	
3	19	19.8%	96	16.0%		31	22.5%	138	15.9%	
≥ 4	78	27.6%	283	47.2%		116	27.2%	427	49.3%	

NS: non-significant.

Regarding adjuvant drugs taken during IVF/ICSI procedure, prednisolone did not affect pregnancy rate in both TAI positive and TAI negative groups (p > 0.05 for both subgroups).

TAI status according to lab tests before IVF/ICSI procedure: TAI status did not statistically differ when comparing different ovarian reserves observed according to basal FSH and AMH. Similar results were found for the TSH <2.5 and the blue age subgroup (Table 3).

**Table 3:** TAI positivity in Low and High Normal TSH subgroups according to pre-cycle tests.

	TSH <2.5					TSH <4.5				
	TAI Positive		Total (% of cycles)		*p* Value	TAI Positive		Total (% of cycles)		*p* Value
Basal FSH and E2 (n = 719)					NS					NS
Normal	78	24.7%	316	64.4%	NS	122	26.6%	459	63.8%	NS
Poor Ovarian Reserve	9	16.4%	55	11.2%		20	27.0%	74	10.3%	
Negative Feedback	32	26.7%	120	24.4%		41	22.0%	186	25.9%	
AMH (n = 580)					NS					NS
Low	51	23.8%	214	54.2%		78	25.7%	304	52.4%	
Normal	51	28.2%	181	45.8%		85	30.8%	276	47.6%	

NS: non-significant.

### 3.1 Main outcomes

The pregnancy rate did not differ statistically between TAI positive and TAI negative groups. Also, no statistical difference was found regarding pregnancy rate when including only male factor infertility and PGD cases together as an IVF/ICSI indication (Table 4).

**Table 4:** Clinical pregnancy rate among TAI positive and negative cycles in different subgroups.

	Pregnant		Total (% of cycles)		Cancelled		*p* Value
All Cycles							NS
TAI Negative	239	38.2%	625	74.4%	72	10.3%	
TAI Positive	97	45.1%	215	25.6%	32	13.0%	
TSH > 2.5							NS
TAI Negative	170	39.0%	436	75.7%	55	11.2%	
TAI Positive	67	47.9%	140	24.3%	22	13.6%	
TSH: 2.5–4.5							NS
TAI Negative	69	36.5%	189	71.6%	17	8.3%	
TAI Positive	30	40.0%	75	28.4%	10	11.8%	
Male Factor and PGD							NS
TAI Negative	111	37.4%	297	66.9%	40	11.9%	
TAI Positive	45	45.9%	98	22.1%	9	8.4%	
Primary Infertility							*p* < 0.05
TAI Negative	206	37.3%	552	66.3%	64	10.4%	
TAI Positive	89	47.1%	189	22.7%	27	12.5%	
IVF History < 2							NS
TAI Negative	147	43.6%	337	65.4%	22	6.1%	
TAI Positive	67	45.9%	146	28.3%	10	6.4%	
Blue Age							NS
TAI Negative	174	42.6%	408	74.0%	27	6.2%	
TAI Positive	67	46.9%	143	26.0%	11	7.1%	
Red Age							NS
TAI Negative	65	30.0%	217	75.1%	45	17.2%	
TAI Positive	30	41.7%	72	24.9%	21	22.6%	
Normal AMH							NS
TAI Negative	77	43.8%	176	70.4%	15	7.9%	
TAI Positive	30	40.5%	74	29.6%	11	12.9%	
Low AMH							NS
TAI Negative	49	26.9%	182	74.6%	43	19.1%	
TAI Positive	23	37.1%	62	25.4%	16	20.5%	
Normal Ovarian Reserve							NS
TAI Negative	110	36.3%	303	74.3%	29	8.7%	
TAI Positive	42	40.0%	105	25.7%	15	12.5%	
Poor Ovarian Reserve							*p* < 0.05
TAI Negative	10	24.4%	41	78.8%	13	24.1%	
TAI Positive	7	63.6%	11	21.2%	9	45.0%	

NS: non-significant.

In addition, there was not any significant correlation between TAI status and pregnancy rate with respect to cases with a history of cycles of less than two. On the other hand, TAI significantly affected the pregnancy rate in only primary infertility cases (p < 0.05) (Table 4).

Furthermore, the pregnancy rate was also not significantly affected by TAI status when comparing TAI positive and TAI negative cases in the subgroups of TSH <2.5, TSH 2.5–4.5, blue and red Age, normal and low AMH and ovarian reserve regarding FSH. Meanwhile, it only affected the pregnancy rate in the poor ovarian reserve subgroup (p < 0.05) (Table 4).

**Fig. 1:**
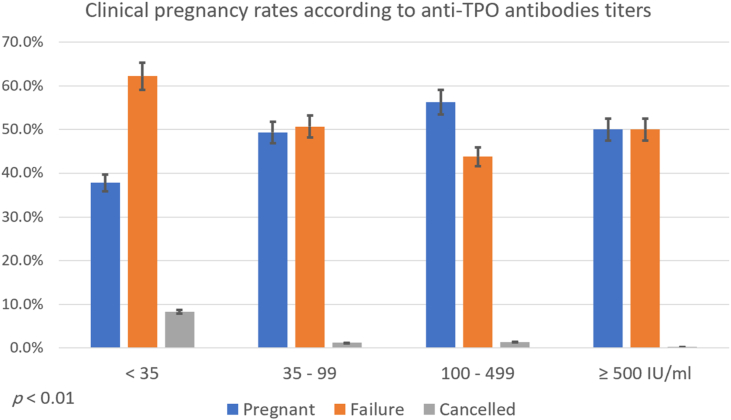
Clinical pregnancy rates according to *anti*-TPO antibodies titers.

**Fig. 2:**
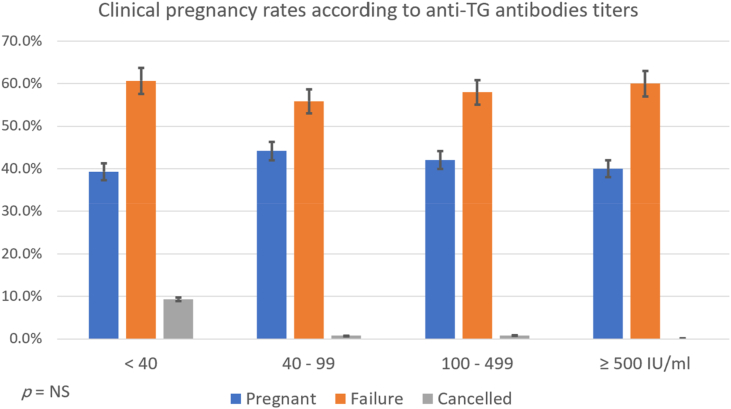
Clinical pregnancy rates according to *anti*-TG antibodies titers.

Finally, clinical pregnancy rate differed significantly between different *Anti*-TPO antibodies titers subgroups (p < 0.05), but not with *anti*-TG antibodies titers subgroups (Fig. 1 and Fig. 2).

## 4 Discussion

About 18–20% of couples of reproductive age have fertility problems. Infertility is defined as not being able to conceive after a year of unprotected intercourse [21,22].

Thyroid hormones interfere with numerous aspects of reproduction. Normal ovarian function and pregnancy outcomes are adversely affected by hypothyroidism and hyperthyroidism [23,24]. Since the early 1990s, many studies about the influence of thyroid autoantibodies on recurrent miscarriages and infertility in euthyroid women have been published [23,24]. They concluded that recurrent abortions and failure to conceive are associated with increased positivity of thyroid autoantibodies [9,25,26]. Additionally, recent systematic reviews emphasized this association in the presence of TAI and the increased number of miscarriages among subfertile women who achieved pregnancy through IVF [27].

This study is an assessment of a large cohort of patients in our region, most baseline characteristics of both groups were highly similar and thorough subanalysis was done, which in turn helped in excluding confounding factors affecting the outcome of interest.

Studies found an increased prevalence of TAI in women referred to fertility clinics compared with the normal population [9,28], previous study by Aljarad et al. in the Syrian population showed the same result with a prevalence of 22.1% [10]. In our study, we found that the prevalence of TAI positivity in our sample was 25.3% in patients undergoing IVF/ICSI at our center. Chai et al. reported that it was 19.7% among women who underwent their first ART in Hong Kong [5] and Sakar et al. reported TAI positivity of 19.52% in Turkey [6]. Such differences may be arising due to heterogeneity of women samples studied, differences due to small sample sizes, differences due to the assays used to detect TAI, and finally differences arising from ethnicity as well as geographical locations, and the role of the highly variable iodine nutritional intake in the populations investigated [8].

Whether to screen or not for thyroid function and TAI regarding fertility care remains debatable [20] and cost-effectiveness of tests remains a crucial factor for low-income countries.

Despite the difference in the prevalence of TAI in our study population, the pregnancy rate did not statistically differ between TAI positive and TAI negative groups. A previous meta-analysis showed the same result [29]. In addition, similar to a previous study [5], a similar result was found in cases with a history of cycles of less than two.

Subgroup analysis of the outcome of clinical pregnancy rates when using a stricter threshold of TSH of 2.5 mIU/L also showed no difference to using an upper limit of 4.5 mIU/L (Table 4), this was also similar to the analysis done in the Unuane et al. paper on cumulative delivery rates [20].

Moreover, to exclude factors induced by female infertility factor as an indication for IVF/ICSI, we conducted the analysis including only male factor and PGD together as an IVF/ICSI indication. The result was also similar with no significant pregnancy outcome difference between TAI positive and negative groups, similar to a previous study published by Tan et al., 2018 [30]. On the other hand, we found that TAI affected pregnancy rates when including only primary infertility cases (p < 0.05). The absence of a significant difference in secondary infertility cases may be due to an additional onset of a secondary disease as the primary etiology. In addition, immune causes may be more prevalent in primary infertility cases [31].

In the presented study, the presence of TAI did not affect the fertilization rate (Table 2) similar to a previous study by Kutteh et al. [32], but contrary to Zhong et al. [13].

Clinical pregnancy rate did not differ statistically between blue and red age subgroups regardless of TAI status. Whereas a study on cumulative delivery rates by Unuane et al. [20]. Showed the same result for different age categories. Although 32.9% of our studied women were more than 40 years of age compared to approximately only 15% in the Unuane et al. [20]. paper.

Pregnant women using prednisolone supplementation during IVF/ICSI procedure were found to have a significantly higher TAI (69.8%, n = 67, p < 0.05). Although in our study this supplementation did not affect pregnancy rates in both TAI groups, a previous study's findings were contrary to ours regarding prednisolone supplementation. There was also a strong association between the presence of thyroid autoantibodies and poor IVF outcomes [33]. More studies showed pregnancy rates were significantly improved in women undergoing IVF with the conjugated use of glucocorticoids [[34], [35], [36]]. Though these findings are applicable to the everyday use of glucocorticoids, the same might not be true for women with autoantibodies [[35], [36], [37]].

### 4.1 Study limitations

We could not include clinical, subclinical hypothyroidism, or central hypothyroidism patients as our data did not include T3, Free T3, T4 or Free T4 Laboratory tests, which were already studied previously. Further studies are needed to include other immunological antibodies such as anti-thyroid stimulating hormone receptors to evaluate their effect on assisted reproductive techniques. Additionally, we could not document long follow up for our studied population in order to detect the effect of TAI positivity status on live birth rate and fetus wellbeing. The benefits of thyroxine supplementation should also be studied in our region-specific population.

Statistical analysis in our large cohort retrospective study controlled most variables possible for the determination of the main outcome. Other small case-control prospective studies resulted in the same outcome [6,38], while others found contrary results [33]. Thus, further large multi-center well-controlled prospective studies are needed to deeply evaluate this outcome.

## 5 Conclusion

The prevalence of TAI in Syrian women having IVF/ICSI is 25.3% which is higher than the normal population. Although several arguments were in favor of the correlation between IVF/ICSI outcomes and Thyroid autoimmune diseases, we concluded that the presence of TAI positivity did not adversely affect the Clinical Pregnancy Rate (CPR), which is the most relevant concern for women undergoing IVF treatment and their physicians. Screening of Thyroid Autoimmunity constitutes a financial burden on patients more than its benefit which is consistent with many previous studies.

## Ethics approval and consent to participate

Ethical approval was obtained from the Ethical and Research Committee in both Faculty of Medicine, Damascus University and Syrian Private University with the approval of Orient Hospital Board of Directors.

## Consent for publication

Not applicable.

## Competing interests

None.

## Data availability

The study data are available from the corresponding author upon reasonable request.

## Funding

None.

## Authors' contributions

All authors contributed to the study concept and design. MA clinically approached the patients. NA, AH, and NN retrospectively checked all patients’ files for the completeness and accuracy of the computerized data before initiating the study. NA and FA conducted the statistical analysis. NA, AH and NN drafted the initial manuscript. MA, HA and NI revised the statistical analysis and the manuscript for scientific accuracy. All authors revised the final version of the manuscript and approved it for publication.

## Provenance and peer review

Not commissioned, externally peer-reviewed.

## Registration of research studies

1. Name of the registry: Impact of Thyroid Autoimmunity in Euthyroid Women on the Outcomes of In Vitro Fertilization
2. Unique Identifying number or registration ID: researchregistry6786
3. Hyperlink to your specific registration (must be publicly accessible and will be checked): https://www.researchregistry.com/register-now#home/registrationdetails/608d54193da41d001dd343fd/

## Guarantor

Ahed Hamed, Nawras Alhalabi.

## List of Abbreviations

AMH: Anti Müllerian Hormone
*Anti*-TG: Anti Thyroglobulin antibodies
*Anti*-TPO: Anti Thyroid Peroxidase antibodies
ART: Assisted Reproduction Techniques
Basal FSH: Basal Follicular Stimulating Hormone
CPR: Clinical Pregnancy Rate
E2: Day 2 Estradiol
ECL: Electrochemiluminescence
ET: Embryo transfer
FRs: Fertilization Rates
GnRH: Gonadotropin-Releasing Hormone
HCG: Human chorionic gonadotropin
HMG: Human Menopausal Gonadotropin
IVF/ICSI: Vitro Fertilization/Intra Cytoplasmic Sperm Injection
IVF: In Vitro Fertilization
IVF-ET: in Vitro fertilization-embryo transfer
PGD: Pre-implantation Genetic Diagnosis
rFSH: Recombinant Follicular Stimulating Hormone
SD: Standard Deviation
T3: Triiodothyronine
T4: Thyroxine
TAI: Thyroid Autoimmunity
TESA: Testicular/Epididymal Sperm Aspiration
TG: Thyroglobulin
TPA: Tripropylamine
TPO: Thyroid peroxidase
TSH: Thyroid Stimulating Hormone
